# The prevalence of malnutrition and impact on patient outcomes among older adults presenting at an Irish emergency department: a secondary analysis of the OPTI-MEND trial

**DOI:** 10.1186/s12877-020-01852-w

**Published:** 2020-11-07

**Authors:** Anne Griffin, Aoife O’Neill, Margaret O’Connor, Damien Ryan, Audrey Tierney, Rose Galvin

**Affiliations:** 1 School of Allied Health, Faculty of Education and Health Sciences, Health Implementation Science and Technology, Health Research Institute, University of Limerick, Limerick, V94 T9PX Ireland; 2 School of Allied Health, Faculty of Education and Health Sciences, Ageing Research Centre, Health Research Institute, University of Limerick, Limerick, Ireland; 3 Department of Ageing and Therapeutics, University Hospital Limerick, Dooradoyle, Limerick, Ireland; 4 Graduate Entry Medical School, Faculty of Education and Health Sciences, University of Limerick, Limerick, Ireland; 5 Retrieval, Emergency and Disaster Medicine Research and Development Unit (REDSPoT), Emergency Department, University Hospital Limerick, Dooradoyle, Limerick, Ireland

**Keywords:** Malnutrition screening, Health and social care professionals, Emergency department, Older adults, Hospital readmission

## Abstract

**Background:**

Malnutrition is common among older adults and is associated with adverse outcomes but remains undiagnosed on healthcare admissions. Older adults use emergency departments (EDs) more than any other age group. This study aimed to determine the prevalence and factors associated with malnutrition on admission and with adverse outcomes post-admission among older adults attending an Irish ED.

**Methods:**

Secondary analysis of data collected from a randomised controlled trial exploring the impact of a dedicated team of health and social care professionals on the care of older adults in the ED. Nutritional status was determined using the Mini Nutritional Assessment- short form. Patient parameters and outcomes included health related quality of life, functional ability, risk of adverse health outcomes, frailty, hospital admissions, falls history and clinical outcomes at index visit, 30-day and 6-month follow up. Aggregate anonymised participant data linked from index visit to 30-days and 6-month follow-up were used for statistical analysis.

**Results:**

Among 353 older adults (mean age 79.6 years (SD = 7.0); 59.2% (*n* = 209) female) the prevalence of malnutrition was 7.6% (*n* = 27) and ‘risk of malnutrition’ was 28% (*n* = 99). At baseline, those who were malnourished had poorer quality of life scores, functional ability, were more frail, more likely to have been hospitalised or had a fall recently, had longer waiting times and were more likely to be discharged home from the ED than those who had normal nutrition status. At 30-days, those who were malnourished were more likely to have reported another hospital admission, a nursing home admission, reduced quality of life and functional decline than older adults who had normal nutrition status at the baseline ED visit. Differences between the MNA SF and 6-month outcomes were similar but not statistically significant.

**Conclusion:**

Over one-third of older adults admitted to an Irish ED are either malnourished or at risk of malnourishment. Malnutrition was associated with a longer stay in the ED, functional decline, poorer quality of life, increased risk of hospital admissions and a greater likelihood of admission to a nursing home at 30 days.

**Trial registration:**

Protocol registered in ClinicalTrials.gov, ID: NCT03739515, first posted November 13, 2018.

## Background

In 2018, the number of people worldwide who were over the age of 65 years outnumbered children who were under the age of five years for the first time in history. It is predicted that this cohort will more than double (from 9 to 16%) by 2050 [1]. In Ireland, census figures from 2011 to 2016 reported that the proportion of adults aged 65 years or older increased by 19.1% [2]. Further projections from the 2016 census predict that the number of adults aged over 65 years will increase significantly from a level of 629,800 to a potential 1.6 million by 2051, with the most dramatic increase occurring among those aged over 80 years [3]. The increasing ageing population along with a higher number of individuals with multi-morbidity are some of the main demographic drivers of incremental increases in Emergency Department (ED) attendances [4, 5]. In Ireland, an increase was observed in the proportion of older adults who visited the ED at least once in the year previous from 15 to 18% during 2009–2016 [6]. For older adults with frailty, the proportion with at least one overnight hospital admission increased (from 23 to 31%) while the average number of nights spent in hospital more than doubled (from 2.7 nights to 6.5 nights) [6].

Protein-energy malnutrition, often referred to simply as malnutrition, is a condition resulting from inadequate intake or an inability to absorb and/or digest adequate energy and/or protein [7]. A strong association between malnutrition and adverse health outcomes among older adults is well documented including increased morbidity and mortality [8–10]. Nutritional vulnerability contributes to more medical complications, longer hospital stays, increased likelihood of nursing home admission and poorer quality of life [11–13]. Total costs associated with malnutrition among institutionalised and community-dwelling older adults are reported as considerably higher than those among well-nourished older adults, predominately due to higher use of health care resources — GP consultations, hospitalisations, health care monitoring, and treatments [14]. Of a limited number of studies investigating malnutrition among older adults presenting at EDs, prevalence rates are reported as 15–29% [15–18] and have been associated with an increase in short-term mortality [16, 19].

Malnutrition screening in the ED can therefore capture a nutritionally vulnerable population that could otherwise be overlooked as not all individuals who attend the ED are admitted to hospital, where screening for malnutrition usually takes place [11, 15]. The Mini Nutrition Assessment is a valid nutritional screening tool recommended for use by the European Society for Clinical Nutrition and Metabolism (ESPEN) guideline on clinical nutrition and hydration in geriatrics [8, 20]. It takes into account physical and mental functional impairments that regularly contribute to the development of malnutrition and thus, considers an existing risk of malnutrition [8, 21].

Given a dearth of information this study aimed to determine the prevalence of malnutrition among older adults from screening on admission to an Irish ED and to report the observed patient outcome factors between categories of malnutrition risk at baseline, 30-days and 6-months follow up.

## Methods

### Study design

This is an observational study from a secondary analysis of a single-centre randomised controlled trial, OPTI-MEND, which aimed to determine and measure the impact of a dedicated health and social care professional (HSCP) team on the quality, safety, timeliness and cost-effectiveness of care of older adults in the ED. The protocol for the trial is published elsewhere [22]. The OPTI-MEND study received ethical approval from the Health Service Executive (HSE) Mid-Western Regional Hospital Research Ethics Committee (ref. 103/18). Written informed consent was obtained from all study participants.

### Participants

Adults aged 65 years or older who presented to the ED at the University Hospital of Limerick (UHL), between December 2018 and May 2019 (inclusive), were considered eligible for the OPTI-MEND study. Inclusion criteria required 1) the capacity (Mini-Mental State Examination ≥17) and willingness to provide informed consent; 2) baseline mobility and functional status; and 3) lower urgency defined as medical stability presenting with any of the complaints presented in Table 1 as per the Manchester Triage System 2–5. The exclusion criteria included: 1) aged under 65 years; 2) medically unstable; 3) neither the patient nor the carer could sufficiently communicate in English to complete consent or baseline assessment; and 4) presentation and discharge outside of HSCP operational hours (8 am to 5 pm, Monday to Friday) [22, 23]. A total of 392 older adults were approached to participate with 38 declining to participate and one exclusion who did not meet the inclusion criteria.

**Table 1:** Presenting complaint as per Manchester Triage System [23]

Before Medical Work-up^a^	***After Medical Work-up***^b^
Limb problems	Chest pain
Falls	Shortness of breath
Unwell adult	Abdominal pain
Back pain	Headache
Urinary problems	
Ear and facial problems	

^a^The health and social care professional (HSCP) team proactively treated these individuals without prior assessment by a physician ^b^The HSCP team awaited medical clearance prior to assessment and intervention

### Tools and procedures

Participants of the OPTI-MEND study [22] underwent a baseline assessment of function and quality of life by one of the dedicated HSCP team (senior physiotherapist, senior occupational therapist and senior medical social worker) or a research nurse. Outcome assessment at follow-up (30-days and 6 months following index visit) was conducted via telephone.

Each participants baseline information included demographic and social details (age, sex, marital and residential status, mode of transport to ED, source of referral), the duration of patient ED stay (mean number of hours from time of arrival to discharge or admission); hospital admissions from the ED (defined as the proportion of patients who are admitted to hospital after their index visits), the duration of hospital admission after the ED index visit, assessment of frailty using the Clinical Frailty Scale [24] and, risk of adverse health outcomes using the Identification of Seniors at Risk (ISAR) [25]. The Mini Nutrition Assessment-Short Form (MNA-SF) was included as an additional measure of assessment to the original OPTI-MEND trial. The MNA-SF allows for the measurement of either body mass index (BMI) or calf circumference enabling use with individuals who are immobile or in situations where weight and height cannot be measured [20]. Previous research has established the criterion validity of this tool in healthcare settings (community, rehabilitation, residential care, and hospital) among older people. The MNA-SF is comprised of six individual components which include documenting food intake, weight loss, mobility, stress or disease, neuropsychological problems and either Body Mass Index (BMI) or calf circumference (CC). It is scored out of a possible total of 14 points with the following categories: 0–7 points: ‘Malnourished’, 8–11 points: ‘at risk of malnourishment’ and 12–14 points: ‘normal nutritional status’.

### Outcome measures

Adverse outcomes recorded from the index visit included ED outcome (admission, discharge home, transfer to other hospital), the hospital length of stay (measured in days), and patient’s length of stay in the ED (measured in hours).

Outcome measures included the number of ED re-attendance, nursing home admissions, unplanned hospital visits (and length of stay) and mortality within 30 days and 6 months of the initial index visit. Healthcare utilisation (visits to a general practitioner (GP), public health nurse, home help, private consultation, outpatient department visit, or allied health services) was captured at 30 days and 6 months after the index visit. Assessment of patient-oriented outcomes included the Barthel Index for Activities of Daily Living [26] as a global measure of functional status and the EuroQoL’s 5- level of the EQ-5D (EQ-5D-5 L) to measure health-related quality of life [27] assessed at baseline and follow-up (30 days and 6 months). At 30-days, functional status was measured by the change in the Barthel index from baseline to 30-days (no change, reduced function, improved function), and change in quality of life (QOL) was measured by the change in EQ-5D (no change, reduced QOL, improved QOL). At 6-months further decline in function and QOL was measured by the change from 30-days to 6-months.

### Statistical analysis

Aggregate anonymised participant data linked from baseline to 30-days and 6-month follow-up were used for statistical analysis. Descriptive statistics of the study participants were conducted. Categorical data were described by counts and percentages. Continuous data that approximated a normal distribution were described using means and standard deviations. Skewed data were described using medians and interquartile ranges. Differences between patient’s demographic, psychological/social, environmental/economic and physiological/biomedical information and the MNA-SF categories were tested using Pearson’s Chi-square test (or Fisher’s exact test if appropriate) for categorical data. For continuous data, differences were tested using the one-way ANOVA test or Kruskal Wallis tests where appropriate. Eta^2^ was used to measure effect size for three or more groups, where 0.01, 0.06 and 0.14 represent a small, medium and large effect. Cramer’s V was used to measure the size of the effect between categorical variables, with V = 0.1, 0.3 and 0.5 for a small, medium and large effect, respectively. Hierarchical logistic regression models were used to further analyse associations between the MNA-SF categories and the observed decline in functional status and quality of life at follow up. A 5% level of significance was used for all statistical tests. All statistical analysis was undertaken using SPSS Version 24.

## Results

### Malnutrition classification

A total of 353 patients participated with a mean age of 79.6 (SD = 7.0) years; 59.2% (*n* = 209) of participants were female. Using the MNA-SF screening tool, 7.6% (*n* = 27) older adults attending the ED were categorised as malnourished, 28.0% (*n* = 99) were categorised as at risk of malnutrition and 64.3% (*n* = 227) had a normal nutritional status. Table 2 presents the distribution of the component information of the MNA-SF tool as recorded in the sample of patients.

**Table 2:** Prevalence (N (%)) of states of nutrition and individual components that contribute to nutrition status according to the MNA-short form screening tool among older adults (*n* = 353) at index visit to the ED

Malnutrition Screening	Criteria	N (%)
**MNA-SF Total**	Malnourished (MNA score 0–7 points)	27 (7.6)
	At risk of malnutrition (MNA score 8–11 points)	99 (28.0)
	Normal nutritional status (MNA score 12–14 points)	227 (64.3)
**Individual components of the MNA-SF tool**		
Food intake	Severe decrease in food intake	13 (3.7)
	Moderate decrease in food intake	59 (16.7)
	No decrease in food intake	281 (79.6)
Weight loss	Weight loss greater than 3 kg	21 (5.9)
	Does not know	17 (4.8)
	Weight loss between 1 and 3 kg	34 (9.6)
	No weight loss	281 (79.6)
Mobility	Bed or chair bound	7 (2.0)
	Able to get out of bed / chair but does not go out	85 (24.1)
	Goes out	261 (73.9)
Stress or disease	Yes	74 (21.0)
	No	279 (79.0)
Neuropsychological problems	Severe dementia or depression	22 (6.2)
	Mild dementia	37 (10.5)
	No psychological problems	294 (83.3)
BMI kg/m^2^ (*n* = 49)^a^	BMI less than 19 kg/m^2^	6 (1.7)
	BMI 19 to less than 21 kg/m^2^	4 (1.1)
	BMI 21 kg/m^2^ to less than 23 kg/m^2^	6 (1.7)
	BMI 23 kg/m^2^ or greater	33 (9.3)
Calf circumference (*n* = 296) ^a^	CC less than 31 cm	50 (14.2)
	CC 31cm or greater	246 (69.7)

^a^Either the BMI (kg/m^2^) or the calf circumference (cm) was used to classify individuals into the MNA categories

*MNA* mini nutritional assessment; *BMI* body mass index; *CC* calf circumference

When asked about the 3 months before ED attendance, weight loss was reported by 15.5% (*n* = 55) older adults with 5.9% (*n* = 21) stating a loss of body weight greater than 3 kg in the previous 3 months. A moderate to severe decline in food intake was reported by 20.4% (*n* = 72) older adults. Measured BMI was recorded for 13.0% (*n* = 49) of the 353 patients, with mean BMI of 27.8 (SD = 5.7) kg/m^2^. Among those with a BMI categorised as overweight and obese (67.4%, *n* = 31), 13% (n = 4) were identified as ‘at risk of malnutrition’ using the MNA-SF with the remainder having normal nutrition status.

### Participant characteristics between MNA-SF categories

Table 3 shows the characteristics of the study participants as lower urgency patients admitted to the ED and the difference in characteristics between MNA-SF categories. There was no difference in the number of participants in the age groups (60–74, 75–84, and 85+ years) between the MNA-SF categories. Results suggest that those who were screened as being malnourished had poorer QOL scores (EQ-5D 15 (6.0) vs 12 (7) and 11 (6.0), *p* < 0.001; EQ-VAS 50 (20) vs 50 (30) and 65 (30) , p < 0.001), were more frail (Clinical Frailty Score 5.6 (1.1) vs 4.5 (3.7) and 3.7 (1.2), p < 0.001), more at risk of adverse health outcomes (ISAR score 3.7 (1.2) vs 2.8 (1.3) and 2.3 (1.2), *p* < 0.001), had poorer functional status (Barthel score 13.0 (9.0) vs 17.0 (6.0) and 18.0 (5.0) , p < 0.001), and longer waiting times in the ED (PET 22.6 (11.4) vs 18.1 (17.2) and 17.2 (14.4) hours, p < 0.001) compared to older adults who were at risk of malnutrition or had normal nutritional status, respectively. Those who had normal nutrition status were less likely to be discharged home from the ED (20.3% (*n* = 46) vs 40.4% (*n* = 40) and 33.3% (*n* = 9) , p < 0.001) compared to older adults who were screened at risk of malnutrition or had malnutrition, respectively.

**Table 3:** Baseline characteristics of patients (n 353) on admission to ED according to nutrition status as categorised by the MNA-SF

			MNA-SF categories			
Characteristics, n (%) or mean (SD) or median (IQR)		Full sample n = 353	Normal nutrition n = 227	At risk of malnutrition n = 99	Malnourished n = 27	***p***-value (effect size)
**Demographic information**						
Sex	Male	144 (40.8)	101 (44.5)	36 (36.4)	7 (25.9)	0.10 (0.11)
	Female	209 (59.2)	126 (55.5)	63 (63.6)	20 (71.4)	
Age	60–74	83 (23.5)	60 (26.4)	21 (21.2)	2 (7.4)	0.19 (0.09)
	75–84	171 (48.4)	108 (47.6)	49 (49.5)	14 (51.9)	
	85+	99 (28.0)	59 (26.0)	29 (29.3)	11 (40.7)	
Marital status	Married	135 (38.2)	94 (41.8)	34 (34.7)	7 (25.9)	0.29 (0.11)
	Single	51 (14.4)	33 (14.7)	14 (14.3)	4 (14.8)	
	Divorced	11 (3.1)	5 (2.2)	6 (6.1)	0 (0.0)	
	Widowed	153 (43.3)	93 (41.3)	44 (44.9)	16 (59.3)	
Residential status	Lives alone	153 (43.3)	103 (45.4)	39 (39.4)	11 (40.7)	0.46 (0.07)
	Lives with family	183 (51.8)	115 (50.7)	55 (55.6)	13 (48.1)	
	Other	17 (4.8)	9 (4.0)	5 (5.1)	3 (11.1)	
Mode of Entry	Ambulance	171 (48.4)	98 (43.2)	57 (57.6)	16 (59.3)	0.17 (0.10)
	Private Transport	177 (50.1)	125 (55.1)	41 (41.4)	11 (40.7)	
	Public Transport / walk in	5 (1.4)	4 (1.8)	1 (1.0)	0 (0.0)	
Source of Referral	GP	124 (35.1)	75 (33.0)	36 (36.4)	13 (48.1)	0.05 (0.12)
	Self-referral	210 (59.5)	140 (61.7)	60 (60.6)	10 (37.0)	
	Other^b^	19 (5.4)	12 (5.3)	3 (3.0)	4 (14.8)	
Presenting Problem	Acute disease / injury (moderate)	317 (89.8)	204 (89.9)	88 (88.9)	25 (92.6)	0.12 (0.10)
	Acute disease / injury (severe)	23 (6.5)	18 (7.9)	5 (5.1)	0 (0.0)	
	Gastro complaint	13 (3.7)	5 (2.2)	6 (6.1)	2 (7.4)	
Triage	Orange	36 (10.2)	25 (11.0)	9 (9.1)	2 (7.4)	0.74 (0.06)
	Yellow	295 (83.6)	187 (82.4)	83 (83.8)	25 (92.6)	
	Green	22 (6.2)	15 (6.6)	7 (7.1)	0 (0.0)	
EQ-5D total^c^		12 (6)	11 (6)	12 (7)	15 (6.0)	**< 0.001 (0.17)**
EQ VAS^c^		60 (30)	65 (30)	50 (30)	50 (20.0)	**< 0.001 (0.19)**
Barthel index^c^		18 (5)	18.0 (5)	17.0 (6.0)	13.0 (9.0)	**< 0.001 (0.32)**
ISAR score^a^		2.52 (1.2)	2.3 (1.2)	2.8 (1.3)	3.7 (1.0)	**< 0.001 (0.32)**
Clinical frailty score^a^		4.1 (1.4)	3.7 (1.2)	4.5 (3.7)	5.6 (1.1)	**< 0.001 (0.41)**
Falls past 3 months	No	164 (46.6)	96 (42.3)	57 (58.2)	11 (40.7)	**0.03 (0.14)**
	Yes	188 (53.4)	131 (57.7)	41 (41.8)	16 (59.3)	
Hospitalised past 6 months	No	239 (67.9)	169 (74.8)	56 (56.6)	14 (51.9)	**0.001 (0.20)**
	Yes	113 (32.1)	57 (25.2)	43 (43.4)	13 (48.1)	
**Outcome of index visit**						
PET^c^		18.1 (15.1)	17.2 (14.4)	18.1 (17.2)	22.6 (11.4)	**< 0.001 (0.22)**
Outcome	Admission	220 (62.3)	162 (71.4)	46 (46.5)	12 (44.4)	**< 0.001 (0.19)**
	Discharge home	95 (26.9)	46 (20.3)	40 (40.4)	9 (33.3)	
	Transfer to other hospital	38 (10.8)	19 (8.4)	13 (13.1)	6 (22.2)	
HoLOS^c^		9.0 (15)	8 (12)	9 (23)	10 (12)	0.21 (0.19)

Statistical difference is reported in bold between categories of nutritional status (*P* ≤ 0.05)

^a^mean (SD) is presented

^b^other includes injury unit, nursing home, Out of Hours clinic, walk in clinic

^c^median (IQR) is presented

*MNA* mini nutritional assessment, *HoLOS* hospital length of stay in days, *PET* patient length of stay in ED in hours, *EQ-5D total* quality of life score, *EQ VAS* visual analogue scale score, *ISAR score* identification of seniors at risk

### Differences in follow-up outcomes between MNA-SF categories

Table 4 presents the outcomes measured at 30-day follow-up since the index ED visit and differences between MNA-SF categories. In general, those who were malnourished were more likely to have reported a hospital admission (29.6% (*n* = 8) vs 12.1% (*n* = 12) and 10.6% (*n* = 24), *p* = 0.02), a nursing home admission (33.3% (*n* = 9) vs 24.4% (n = 24) and 8.4% (*n* = 19), *p* < 0.001), a reduced quality of life (40% (*n* = 10) vs 15.1% (*n* = 13) and 13.3% (*n* = 28), p = 0.02) and reduced functional status (52% (n = 13) vs 36% (*n* = 31) and 24.8% (*n* = 52), p = 0.02), compared to the older adults who were at risk of malnutrition or had normal nutritional status, respectively.

**Table 4:** Differences in reported patient outcomes across MNA-SF categories among 353 older adults at 30-day follow-up from index visit in an Irish ED

Patient outcome, n (%)		Full Sample	MNA categories			
			Normal nutrition (n = 227)	At risk of malnutrition (n = 99)	Malnourished (n = 27)	P-value (Effect size)
Functional decline	No change	141 (43.9)	96 (45.7)	39 (45.3)	6 (24.0)	**0.02 (0.14)**
	Reduced function	96 (29.9)	52 (24.8)	31 (36.0)	13 (52.0)	
	Improved function	84 (26.2)	62 (29.5)	16 (18.6)	6 (24.0)	
QOL decline	No change	47 (13.3)	32 (15.2)	13 (15.1)	2 (8.0)	**0.02 (0.14)**
	Reduced QOL	51 (15.9)	28 (13.3)	13 (15.1)	10 (40.0)	
	Improved QOL	223 (69.5)	150 (71.4)	60 (69.8)	13 (52.0)	
ED revisit	No	296 (83.9)	189 (83.3)	86 (86.9)	21 (77.8)	0.48 (0.06)
	Yes	57 (16.1)	38 (16.7)	13 (13.1)	6 (22.2)	
Number of ED visits	0	298 (84.4)	191 (84.1)	86 (86.9)	21 (77.8)	0.49 (0.08)
	1	43 (12.2)	27 (11.9)	10 (10.1)	6 (22.2)	
	2+	12 (3.4)	9 (4.0)	3 (3.0)	0 (0.0)	
Hospital admissions	No	309 (87.5)	203 (89.4)	87 (87.9)	19 (70.4)	**0.02 (0.15)**
	Yes	44 (12.5)	24 (10.6)	12 (12.1)	8 (29.6)	
HoLOS at revisit^a^		8.4 (4.7)	8.7 (4.9)	7.3 (4.2)	9.3 (5.7)	0.46 (0.21)
Healthcare use	No	110 (31.2)	58 (25.6)	42 (42.4)	10 (37.0)	**0.008 (0.17)**
	Yes	243 (68.8)	169 (74.4)	57 (57.6)	17 (63.0)	
Frequency of healthcare use^b^		1 (2)	1 (2)	1 (1)	1 (1)	0.69 (0.05)
Nursing home admission	No	301 (85.3)	208 (91.6)	75 (75.8)	18 (66.7)	**< 0.001 (0.25)**
	Yes	52 (14.7)	19 (8.4)	24 (24.2)	9 (33.3)	

Statistical difference is reported in bold between categories of nutritional status (p ≤ 0.05)

^a^mean (SD) is presented

^b^median (IQR) is presented

*MNA-SF* mini nutritional assessment,-short form *QOL* quality of life, *ED* emergency department, *HoLOS* hospital length of stay

While similar patterns were observed at 6 months, with those categorised as being malnourished most at risk of functional decline, these differences between the MNA- SF and 6-month outcomes were not statistically significant (Appendix in Table 7).

### Malnutrition status as a predictor of reported decline in functional status and quality of life

Hierarchical logistic regression models were used to analyse associations between MNA-SF categories of nutritional status and the reported decline in functional status and quality of life at 30-day follow up. Table 5 shows the models of declining functional status and MNA-SF categories controlling for sex, age, quality of life, risk of adverse health outcomes and frailty. The patients who were screened as malnourished in the ED were over three times more likely to have functional decline at follow-up, when compared to the patients who were identified as having a normal nutritional status in the ED (Model 1; unadjusted OR of 3.29 (95% CI = 1.41, 7.66), *p* = 0.008). However, when controlling for sex, age, quality of life, risk of adverse health outcomes and frailty, malnutrition was no longer a significant predictor of functional decline (Model 3; unadjusted OR of 1.53 (95% CI = 0.57, 4.13,) *p* = 0.61).

**Table 5:** Logistic regression model of decline in functional status (measured as Barthel Index) among older adults at 30-day follow up from index visit to the ED”

		30-day follow up					
		Model 1		Model 2		Model 3	
		OR (95% CI OR)	p-value	OR (95% CI OR)	p-value	OR (95% OR)	p-value
**MNA categories**	**Normal nutritional status**	1	0.008	1	0.02	1	0.61
	**At risk of malnutrition**	1.71 (1.00, 2.94)		1.71 (0.98, 3.00)		1.27 (0.68, 2.36)	
	**Malnourished**	3.29 (1.41, 7.66)		3.08 (1.28, 7.39)		1.53 (0.57, 4.13)	
**Sex**	**Male**			1	0.12	1	0.07
	**Female**			0.67 (0.40, 1.11)		0.61 (0.36, 1.01)	
**Age**	**60–74**			1	< 0.001	1	0.002
	**75–84**			2.98 (1.36, 6.51)		3.50 (1.53, 8.01)	
	**85+**			5.34 (2.33, 12.22)		5.20 (2.11, 12.78)	
**EQ-5D score**						1.08 (1.01, 1.15)	0.04
**EQ VAS score**						0.99 (0.98, 1.01)	0.31
**ISAR score**						0.92 (0.68, 1.23)	0.56
**Clinical Frailty score**						1.38 (1.03, 1.85)	0.03

Statistical significance if p ≤ 0.05

Model 2 controlling for sex and age

Model 3 controlling for sex, age, quality of life (EQ-5D and EQ-VAS), risk of adverse health (ISAR score) and frailty (CFI score)

*OR* odds ratio, *CI* confidence interval, *MNA* mini nutritional assessment, *EQ-5D total* quality of life score, *EQ VAS* visual analogue scale score, *ISAR score* identification of seniors at risk

Table 6 shows the models of reported decline in quality of life at 30-days follow up and MNA-SF categories controlling for sex, age, functional ability, risk of adverse health outcomes and frailty. Patients who were identified as malnourished in the ED, were over four times more likely to report a decline in quality of life when compared to patients who were identified as having a normal nutritional status (Model 1; unadjusted OR of 4.33 (95% CI = 1.77, 10.59), *p* = 0.005). Furthermore, patients are more likely to report a significant decline in quality of life when confounding factors including sex, age, functional status and frailty are accounted for (Model 3; adjusted OR of 3.66 (95% CI = 1.27, 10.56), *p* = 0.02).

**Table 6:** Logistic regression model of decline in quality of life (EQ-5D) among older adults at 30-day follow up from index visit to the ED

		30-day follow up					
		Model 1		Model 2		Model 3	
		OR (95% CI OR)	p-value	OR (95% CI OR)	***p***-value	OR (95% CI OR)	p-value
**MNA-SF categories**	**Normal nutritional status**	1	0.005	1	0.008	1	0.02
	**At risk of malnutrition**	1.16 (0.57, 2.36)		1.14 (0.55, 2.36)		1.00 (0.46, 2.15)	
	**Malnourished**	4.33 (1.77, 10.59)		4.32 (1.70, 10.95)		3.66 (1.27, 10.55)	
**Sex**	**Male**			1	0.08	1	0.07
	**Female**			0.57 (0.30, 1.08)		0.55 (0.29, 1.05)	
**Age**	**60–74**			1	0.03	1	0.05
	**75–84**			3.28 (1.09, 9.90)		3.11 (1.02, 9.47)	
	**85+**			4.82 (1.54, 15.11)		4.42 (1.37, 14.24)	
**Barthel Index**						1.10 (0.71, 1.45)	0.12
**ISAR score**						1.01 (0.71, 1.45)	0.94
**Clinical Frailty score**						1.33 (0.91, 1.95)	0.14

Statistical significance if *p* ≤ 0.05

Model 2 controlling for sex and age

Model 3 controlling for sex, age, functional ability (Barthel Index), risk of adverse health (ISAR score) and frailty

*OR* odds ratio, *CI* confidence interval, *MNA-SF* mini nutritional assessment-short form, *ISAR score* identification of seniors at risk

## Discussion

This is the first Irish study to screen for malnutrition in a cohort of older adults presenting to a large urban ED. We also provide observations of impact on patient outcomes between categories of nutrition status. Over one in three older adults defined as ‘lower urgency’ on admission were at risk of malnutrition or categorised as malnourished. These older adults were more likely to be frail and at risk of adverse health outcomes, to have experienced a fall in the previous three months, had hospital admissions in the previous six months and reported a poorer functional status and quality of life and spent more hours in the ED. Older adults screened as malnourished at the index ED visit were found to more frequently report decline in functional status and quality of life,  and subsequent hospital and nursing home admissions at 30-day follow up compared to those categorised with a normal nutrition status. Furthermore, malnourished older adults were over three times more likely to report a significant decline in their quality of life at 30-days than those categorised with normal nutrition status independent of age, sex, risk of adverse health outcomes, frailty and functional status.

The number of older people (*n* = 27, 7.6%) found to be malnourished in our study could be described as small. However, we also report a prevalence of risk of malnutrition among older adults (*n* = 99, 28%) representing over a third of the admissions to the ED of lower urgency, medically stable, and cognitively intact older adults as nutritionally vulnerable. A decrease in food intake and weight loss among one in five participants was reported for the three months previous to admission in the ED (Table 2). A continuum of nutritional vulnerability among older adults has previously been described with subjects at risk considered likely to develop malnutrition in the near future [11, 21]. Prevalence rates of malnutrition in ED settings in the USA and Australia have been reported as 12–16% [15, 17, 18, 29]. However, a point of difference between the current study and these other studies is that screening for malnutrition was conducted by research assistants or dietitians trained in nutrition screening and that may affect the accuracy in identifying patients with malnutrition risk [21, 28, 30]. Our study could be described as pragmatic in nature reflecting the realities in clinical practice that nutrition screening may be carried out by clinical staff who are not trained in nutritional assessment.

Screening for malnutrition is an important step in recognising and identifying risk of or diagnosis of malnourishment [8]. However, it is often not completed owing to perceived barriers of screening implementation in hospital settings including time, competence and resources [31, 32]. Most of the studies advocating for screening, do so at the ward level, thus potentially missing those who present via ED and are not admitted to the ward [15]. The estimated cost of care for patients with malnutrition in Ireland has been estimated at €1.4 billion, representing 10% of total healthcare costs with most of the cost (70%) arising in the acute hospital or residential care settings [33]. A recent budget impact analysis has indicated that the implementation of hospital inpatient guidelines for nutrition screening and use of oral nutrition supplements could produce a net cost saving from reduced length of stay by an average of 13.9% in malnourished patients [34]. Malnutrition on admission develops in the community and is likely to be largely unaffected by the implementation of screening programmes in the acute sector [34]. Our study would indicate that the ED should be included in nutrition screening programmes as an earlier opportunity to identify and intervene given the reported increase in hospital and healthcare use and admission observed among older adults screened as malnourished.

A more recent and rapidly growing phenomenon is malnutrition concomitant with obesity in older adults [11]. Our study reflected the realities of clinical practice in the ED and body weight and height were measured when the equipment was available and it was feasible to remove the patient from the bed. Therefore, only forty-nine participants had measurements of weight and height taken to measure BMI despite the majority indicating an ability to get out of bed/chair. Nonetheless, 13% (*n* = 4) of those who were classified as overweight/obese based on BMI were at risk of malnutrition. If BMI was relied upon as a sole indicator of nutrition, it would fail to identify nutritional issues in these individuals. This has also been reported in other studies highlighting that older people can be at nutritional risk although they may be overweight or obese [8, 35, 36].

Any approach to managing nutritional risk needs to be multi-faceted and include the management of co-morbidities, the provision of home and social supports to encourage and facilitate food intake and the implementation of dietary modifications to improve diet quality [35]. Intervention coordinated by an ED registered dietitian providing nutrition support to older adults identified at nutritional risk at presentation to a hospital setting may lead to improved patient outcomes [15]. Consistent with this, interventions designed to improve and sustain optimal nutritional status can also lead to significant improvements in quality of life, for both physical and mental aspects [13, 21].

### Limitations

This study has several limitations. We did not perform a test of the inter-rater reliability of the administration of the MNA-SF, which has not been validated in the ED, although it has been validated in older adults across a variety of settings [20]. A finding from this study was the high proportion of older adults categorised as having a risk of malnutrition (*n* = 40, 40.4%) or malnourished (*n* = 9, 33.3%) that were discharged from the ED. As this study was observational and nutritional screening was conducted by staff who were not trained in diagnosing malnutrition we surmise that the need for further intervention was not recognised. It is previously reported that clinical staff can miss malnutrition in older adults due to a lack of routine nutritional screening in many hospitals [17, 21, 37], and a belief by clinical staff that individual judgement of nutritional status is more superior to screening [38, 39].

Older adults who presented to the ED outside of core HSCP working hours (8 am to 5 pm, Monday to Friday) were not included in this study and therefore it is possible that eligible participants were missed. Also, older adults who were medically unstable or found to be moderately or severely cognitively impaired were excluded from the study. These groups represent a subgroup of older adults that are likely to be at a high risk of malnutrition as a consequence of reduced dietary intake in combination with the effects of catabolic disease [8, 12]. The prevalence of malnutrition among older adults is known to increase with deteriorating functional and health status [21]. We do not know from this study if the observed prevalence of malnutrition was a cause or consequence of the reported decline in quality of life and adverse patient outcomes at follow-up. However, the increased association would indicate the need for further exploration as the assessment of quality of life is recognised as a clinically relevant outcome measure when evaluating intervention in patient populations, particularly older adults [13].

## Conclusions

This study provides preliminary data that over one in three older adults presenting to an Irish emergency department were at risk of malnutrition or classed as malnourished and more likely to suffer adverse outcomes at follow up including declining health and an increased use of health care services. The findings add support to the prioritisation of nutritional screening in clinical practice and public health policy for older adults, particularly targeted towards high risk groups with frailty and multi-morbidity, and at increased risk of functional decline. Further research on a larger sample is recommended to confirm that findings from this study and future research to assess the feasibility and value of integrating ED-based dietetic intervention and impact on patient outcomes among this high risk group with malnutrition.

## Data Availability

The OPTIMEND dataset is currently not publicly availably but any queries can be directed to the senior author and guarantor of the manuscript.

## Appendix

**Table 7:** Differences in reported patient outcomes across MNA-SF categories among 353 older adults at 6 month follow-up from index visit in an Irish ED. There were no differences between the MNA categories and the 6-month outcomes

Differences in 6-month follow-up outcomes by MNA category						
		Full Sample (n = 353)		MNA categories		
			Normal nutrition (n = 227)	At risk of malnutrition (n = 99)	Malnourished (n = 27)	p-value (Effect size)
Functional decline	No change	136 (44.9)	98 (48.5)	31 (39.7)	7 (30.4)	0.05 (0.13)
	Reduced function	94 (31.0)	56 (27.7)	25 (32.1)	13 (56.5)	
	Improved function	73 (24.1)	48 (23.8)	22 (28.3)	3 (13.0)	
QOL decline	No change	76 (25.2)	57 (28.2)	17 (22.1)	2 (8.7)	0.07 (0.12)
	Reduced QOL	85 (28.1)	57 (28.2)	24 (31.2)	4 (17.4)	
	Improved QOL	141 (46.7)	88 (43.6)	36 (46.8)	17 (73.9)	
ED revisit	No	221 (63.3)	150 (66.7)	53 (54.6)	18 (66.7)	0.11 (0.11)
	Yes	128 (36.7)	75 (33.3)	44 (45.4)	9 (33.3)	
Number of ED visits	0	221 (63.3)	151 (67.1)	52 (53.6)	18 (66.7)	0.16 (0.10)
	1	90 (25.5))	54 (24.0)	31 (32.0)	5 (18.5)	
	2+	38 (10.9)	20 (8.9)	14 (14.4)	4 (14.8)	
Hospital admissions	No	258 (73.9)	173 (76.9)	66 (68.0)	19 (70.4)	0.23 (0.09)
	Yes	91 (26.1)	52 (23.1)	31 (32.0)	8 (29.6)	
HoLOS at revisit^a^		12 (19)	9 (20.0)	13 (17.5)	8.5 (64.25)	0.51 (0.15)
Healthcare use	No	78 (22.3)	40 (17.8)	25 (25.8)	13 (48.1)	0.001 (0.20)
	Yes	271 (77.7)	185 (82.2)	72 (74.2)	14 (51.9)	
Frequency of healthcare use^a^		5 (8)	7 (9.0)	3.5 (4.0)	3 (9.5)	0.72 (0.05)
Nursing home admission	No	335 (96.0)	216 (96.0)	93 (95.9)	26 (96.3)	0.99 (0.01)
	Yes	14 (4.0)	9 (4.0)	4 (4.1)	1 (3.7)	

^a^median (IQR) is presented

*MNA* mini nutritional assessment, *QOL* quality of life, *ED* emergency department, *HoLOS* hospital length of stay

## Abbreviations

BMI: Body Mass Index
CC: Calf Circumference
CFS: Clinical Frailty Score
ED: Emergency Department
EQ-5D-5L: EuroQoL Health Related Quality of Life instrument
EQ-Vas: EuroQoL Visual Analogue Scale
ESPEN: European Society for Parenteral and Enteral Nutrition
HSE: Health Service Executive
HoLOS: Hospital Length of Stay
HSCP: Health and Social Care Professional
ISAR: Identification of Seniors At Risk
MNA: Mini Nutritional Assessment
PET: Patient length of stay in the emergency department
QOL: Quality of Life
